# Comparison of Immune Responses Elicited by SARS-CoV-2 mRNA and Recombinant Protein Vaccine Candidates

**DOI:** 10.3389/fimmu.2022.906457

**Published:** 2022-05-19

**Authors:** Yixin Wu, Huicong Zhang, Liuxian Meng, Fusheng Li, Changyuan Yu

**Affiliations:** ^1^College of Life Science and Technology, Beijing University of Chemical Technology, Beijing, China; ^2^Research Department, Sysvax Inc, Beijing, China; ^3^Vaccine Division, Sun Yat-sen Biomedical Institute Limited, Hong Kong, China

**Keywords:** SARS-CoV-2, protein vaccine, mRNA vaccine, IgG subclass antibodies, cellular immune response

## Abstract

After the outbreak of COVID-19, billions of vaccines with different types have been administrated, including recombinant protein vaccines and mRNA vaccines. Although both types of SARS-CoV-2 vaccine can protect people from viral infection, their differences in humoral and cellular immune responses are still not clearly understood. In this study, we made a head-to-head comparison between an mRNA vaccine candidate and a recombinant protein vaccine we developed previously. Results demonstrated that both vaccine candidates could elicit high specific binding and neutralizing antibody titers in BALB/c mice, but with bias towards different IgG subtypes. Besides, the mRNA vaccine candidate induces higher cellular immune responses than the recombinant protein vaccine. To date, this is the first reported study to directly compare the immune responses of both arms between SARS-CoV-2 mRNA and recombinant vaccines.

## Introduction

SARS-CoV-2 becomes one of the most severe health crises in human history. To date, it has caused over 497 million cases including more than 6 million deaths. According to WHO, since it was first reported in December 2019, more than 20 SARS-CoV-2 variants has emerged, and the virus is still mutating. Until now, there have been two variants, Delta and Omicron, listed as Variants of Concern (VOC).

SARS-CoV-2 is an enveloped, single-stranded RNA virus. Its virions are spiral capsids consisting of nucleocapsid (N) proteins bound to the RNA genome, and an envelope composed of membranes (M), envelopes (E), and spike (S) proteins, which can be cleaved into S1 and S2 subunits by proteases. In the infection cycle, the S protein binds to angiotensin-converting enzyme 2 (ACE2), *via* the receptor-binding domain (RBD) at S1 subunit, and then the S2 subunit mediates viral cell membrane fusion by forming a six-helical bundle *via* the two-heptad repeat domain (1, 2). Hence, in the research and development of SARS-CoV-2 vaccines, the full-length S protein, S1, and RBD have been widely researched as potential targets for inducing robust neutralizing antibodies and T cell-mediated immunity (3–5).

After the outbreak, scientists all over the world were devoted to the research of SARS-CoV-2 vaccines. It has been reported that there are 349 vaccine candidates in clinical or pre-clinical development. Among them, 51 are subunit protein vaccines, which are considered safe and simple to produce. In our previous work, we have also developed an RBD recombinant protein vaccine adjuvanted by innovative delivery of poly I:C for stronger immune responses. In the study, poly I:C was first packed with cationic polymer, poly-L-lysine (PLL), and then poly I:C-PLL, as a polyplex core, was loaded into a lipid shell, consisting of DOTAP, cholesterol, DSPC and DMG-PEG_2000_. Results demonstrated that this recombinant RBD protein induces strong neutralizing antibody responses and protects mice from SARS-CoV-2 infection.

At the same time, the success of Moderna’s mRNA-1273 and BioNTech’s BNT162b2 have led to the outbreak in mRNA vaccine research. One of the important benefits of mRNA vaccine is its ability to be scaled up in a fairly short period of time, which is highly beneficial for SARS-CoV-2 with a fast mutation rate. Compared with recombinant protein vaccine, the manufacture of mRNA does not need laboring and expensive cell culture and purification steps. When viral antigen sequences are available, the clinical-scale mRNA vaccines can be rapidly developed and manufactured in a short time period (4). However, current mRNA vaccine has its own shortcomings. It has been reported that BNT162b2 needed to be stored at -80°C for quality control and needed to be shipped in special freezers from corporate centers in Michigan and Wisconsin to distribution centers across the country, and then to designated vaccination centers and individuals. Every step requires diligent care and coordination. The requirements for mRNA-1273 are simpler, but the storage at -20°C also makes shipping and storage a challenge. The two-week interval required for the second dose of the two vaccines also hamper the widespread vaccination (6).

Despite the extensive efforts on developing recombinant protein and mRNA vaccines, their differences in inducing immunity are less explored. In this study, we constructed an mRNA vaccine candidate against SARS-CoV-2 variant B.1.617.2 and made a direct comparison of immune responses with a SARS-CoV-2 RBD recombinant protein vaccine we developed in our previous work. Although both vaccine candidates elicit similar level of humoral responses in BALB/c mice, the superiority of mRNA vaccine in inducing higher cellular immune responses makes it better vaccine candidate for protecting SARS-CoV-2 infection.

## Results

### mRNA Vaccine Delivers RBD Expression Both *In Vitro* and *In Vivo*

**Figure 1:**
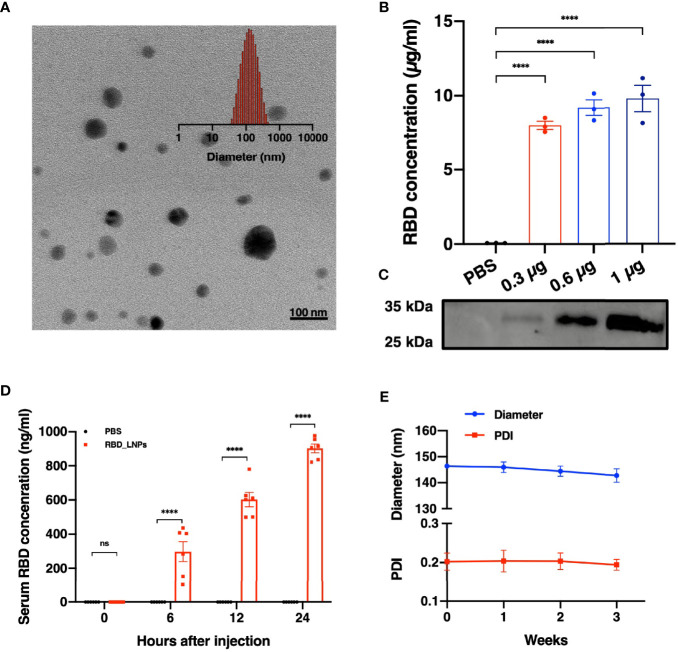
Characterization, expression *in vitro* and *in vivo* delivery of RBD mRNA vaccine. **(A)** TEM and DLS results of RBD_LNPs. **(B)** RBD expression in HEK293T cells. Cells were transfected with RBD_LNPs at different amount of mRNA (0.3µg, 0.6µg, and 1µg/10^6^ cells). The concentration was measured by ELISA at 72 h after transfection. **(C)** HEK293T cells were transfected with RBD_LNPs and western blot of cell supernatant was performed at 72 h after transfection. **(D)** Expression of RBD in mice at different time point after injection. **(E)** DLS results of stability test of RBD_LNPs, which were stored at 4°C for three weeks and the diameter and PDI were evaluated by DLS. The data are presented as mean ± SEM. Significance was calculated using unpaired t test (ns, not significant; *p < 0.05, **p < 0.01, ***p < 0.001, ****p < 0.0001).

Lipid nanoparticles (LNPs) are of great promise and have been widely used in mRNA delivery (7–9). In this study, we chose the RBD of SARS-CoV-2 B.1.617.2 variant as the target antigen and constructed a vaccine, namely RBD_LNPs, which is based on LNPs encapsulating the modified RBD-encoding mRNA. Dynamic light scattering (DLS) and transmission electron microscopy (TEM) were performed to characterize RBD_LNPs. As shown in **Figure 1A**, the average particle size of RBD_LNPs was 144.76 nm, and TEM analysis demonstrated its formation and structure. HEK 293T cells were incubated with RBD_LNPs at different mRNA concentration for 48 h, and cell supernatant was collected to quantify the expression of recombinant RBD protein by ELISA. Results showed that at the minimum dose of 0.3 µg mRNA, the concentration of RBD in supernatant was 8.01 µg/ml (**Figure 1B**). Immunoblotting was also performed to verify the expression of RBD protein (**Figure 1C**). To further test the *in vivo* expression of this mRNA vaccine, RBD_LNPs were injected into BALB/c mice intramuscularly at 1 mg/kg, and the serum was collected at different time point to quantify the expression of RBD by ELISA. Results demonstrated that at 6 h after injection, the expression of RBD was readily detectable, and it was enhanced with the increase of the treatment time. 24 h after injection, the average concentration of RBD in serum reached 901.8 ng/ml (**Figure 1D**), indicating that RBD_LNPs can express RBD protein in mice successfully. Furthermore, RBD_LNPs were stored at 4°C for three weeks, and the size was monitored by DLS. No significant change in the diameter was found (**Figure 1E**).

### mRNA and Recombinant Protein Vaccines Elicited Strong Humoral Responses With Bias Towards Different Subtypes

**Figure 2:**
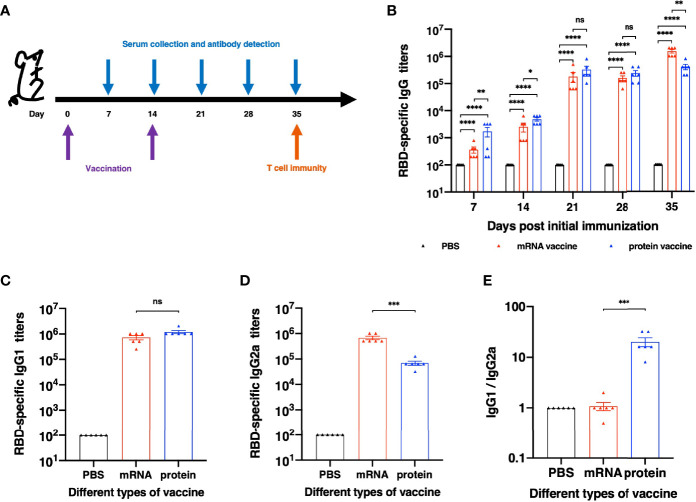
Humoral Immune Response results of different vaccines. **(A)** Schematic diagram of immunization and sample collection schedule. **(B)** SARS-CoV-2-specific IgG antibody titers. **(C)** SARS-CoV-2 RBD-specific IgG1 (Th2) titers and **(D)** IgG2a (Th1) titers of each vaccination group. **(E)** The ratios between specific IgG1 and IgG2a antibody responses. The antibody titers were expressed as the endpoint dilutions that remain positively detectable. The data are presented as mean ± SEM from six mice in each group. PBS was included as the control. Significance was calculated using unpaired t test (ns, not significant; *p < 0.05, **p < 0.01, ***p < 0.001, ****p < 0.0001).

To further evaluate the immunogenicity of this mRNA vaccine and compare it with RBD recombinant protein vaccine candidate, female BALB/c mice were divided into three groups randomly. All mice were immunized and boosted with the same vaccine candidate on day 14. Serum for antibody assays were collected on day 7,14,21,28 and 35 after the first immunization (**Figure 2A**). As shown in **Figure 2B**, after the first immunization, both mRNA and protein vaccine induced detectable RBD-specific immunoglobulin G (IgG) antibodies. Much higher titers were observed after the second vaccination. We next evaluated the ability of both vaccine candidates to induce specific IgG subtype antibodies with sera collected on Day 28 after the initial immunization. As shown in **Figure 2C**, both mRNA and protein vaccines could induce strong RBD-specific IgG1 antibody, and no significant differences were observed between these two groups. As for RBD-specific IgG2a antibody (**Figure 2D**), mRNA vaccine elicits significantly stronger immune response than protein vaccine. The analysis of Th1/Th2 antibody response demonstrated that mice vaccinated with protein vaccine exhibited specific Th2-biased (IgG1) IgG antibody responses (**Figure 2E**).

### mRNA and Recombinant Protein Vaccines Induce Similar Neutralizing Antibodies in BALB/c Mice

**Figure 3:**
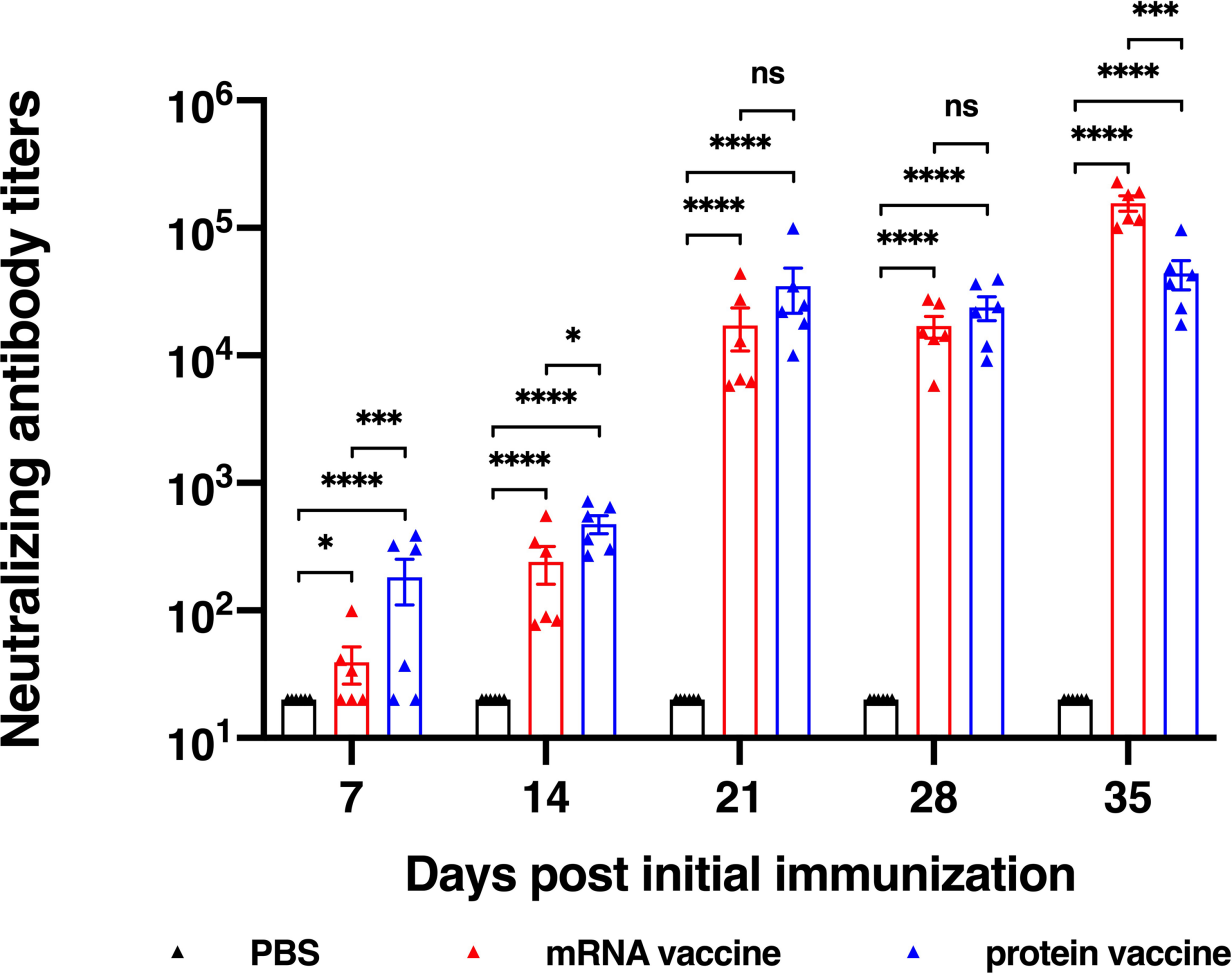
Neutralization ability determined using SARS-CoV-2 pseudovirus. The data are presented as mean± SEM from six mice in each group. PBS was included as the control. Significance was calculated using unpaired t test (ns, not significant; *p < 0.05, **p < 0.01, ***p < 0.001, ****p < 0.0001).

For evaluation of a vaccine efficacy, neutralizing antibody titer is an important factor to be considered, for it is critical for the clearance of virus *in vivo*. In this research, we studied the *in vitro* neutralizing antibody titers of two vaccine candidates. Results showed that both vaccine candidates induce high neutralization antibody titers after second vaccination (**Figure 3**). It is noted that after the first immunization, the protein vaccine elicited higher neutralizing antibody titer than the mRNA vaccine, while on the day 35 after the initial vaccination, neutralizing antibody titer of mRNA vaccine group reached a significantly higher level than that of protein vaccine group, which was similar to the induction of RBD-specific IgG antibody. Together, both mRNA and protein vaccine tested in this study induce high neutralizing antibody titers in mice but with some differences in response dynamics.

### mRNA Vaccine Induces Stronger SARS-CoV-2-Specific Cellular Responses

**Figure 4:**
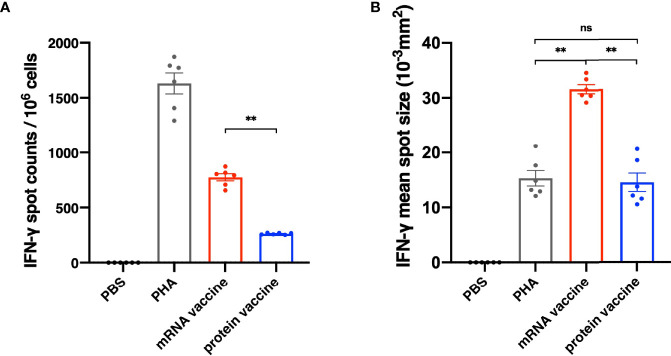
Cellular Immune Response results of different vaccines. **(A)** The number of IFN-γ spot counts per 10^6^ splenocytes. **(B)** IFN-γ mean spot size of different vaccine groups. The data are presented as mean± SEM from six mice in each group. PBS was included as the control. Significance was calculated using unpaired t test (ns, not significant; *p < 0.05, **p < 0.01, ***p < 0.001, ****p < 0.0001).

It has been reported that the antibodies in serum of the recovered patients of SARS vanished in one year (10, 11), while the T cells have existed in the patients for more than six years (12, 13), indicating that cell immunity should be considered in designing SARS-CoV-2 vaccine. On day 35 after the initial vaccination, spleens of mice were harvested and secretion of interferon γ (IFN-γ) in splenocytes was assessed through an ELISpot assay. As shown in **Figure 4A**, the number of IFN-γ-secreting RBD-specific T cells in mRNA vaccine was significantly higher than that in protein vaccine group. In addition to spot counts, IFN-γ spot size should also be considered for illustrating the strength of a single cell to secrete certain cytokines after stimulation. Here, we have found that in the mRNA vaccine group, the average size of IFN-γ spots was significantly larger than the protein vaccine group, as well as the positive control (**Figure 4B**). Taken them together, these results suggested that mRNA vaccines seem better in eliciting cellular immune responses.

## Discussion

After the outbreak of COVID-19, billions of vaccines with multiple types have been administrated, including protein vaccine and mRNA vaccine. Although both vaccine types can protect people from infection, their differences in humoral and cellular immune responses are still not clearly understood. In this study, we constructed an mRNA vaccine against SARS-CoV-2 Delta variant and compared it with a recombinant protein vaccine we studied in our previous work.

Modified RBD-encoding mRNA was loaded in the LNPs containing DOPE, cholesterol, DMG-PEG_2000_ and an ionizable lipid, DLin-MC3-DMA. Two doses of vaccination with RBD_LNPs induced high specific and neutralizing antibody titer and induce robust T cell immune response as well. These results clearly show the superiority of mRNA vaccine over recombinant protein vaccine for inducing virus-specific immune responses, especially for T-cell responses. However, a major concern for mRNA vaccine is its low stability. To test the stability of our mRNA vaccine candidate, Gluc_LNPs were constructed to verify the stability of this mRNA vaccine platform (**Figure S1**). Results showed that even after the storage at 4°C for three weeks, this mRNA_LNPs could still be effective and express certain protein *in vivo*, indicating that this platform would be a promising strategy for mRNA vaccine development.

To compare the immunogenicity of mRNA and protein vaccines, we first evaluated the level of antibody titers, including RBD-specific IgG titers and *in vitro* neutralizing antibody titers. Both vaccines induced detectable RBD-specific IgG and neutralizing antibodies after the first immunization and elevated much higher after the second vaccination. It is noted that on the day 35 after the initial vaccination, neutralizing antibody titer of mRNA vaccine group reached a significantly higher level than that of protein vaccine group. The antibody titers of protein reached the highest level on day 21 after the first vaccination, while that of mRNA vaccine peaked on the day 35 after the first immunization. To conclude, the protein vaccine could elicit humoral immune response faster than the mRNA vaccine, and it takes longer time for mRNA vaccine to reach its peak responses.

Besides the neutralizing activities, antibodies are capable of mediating host effector functions and facilitating the clearance of pathogens from the host (14). In particular, the Fc portion of IgG2a antibodies mediate a high-affinity interaction with activatory Fc receptors and complement components, which can potently trigger Fc receptor-mediated effector functions, including the stimulation of antibody-dependent cell-mediated cytotoxicity and opsonophagocytosis by macrophages (15, 16). The Fc portion of IgG1 antibodies, however, could not interact with activatory Fc receptors so effectively and does not stimulate Fc receptor-mediated immune responses as well (17, 18). Hence, we evaluated the IgG1 and IgG2a antibody titers of each vaccine in this study. It has found that both vaccines can elicit high IgG1 antibody titers and the mRNA vaccine had induced significantly higher IgG2a titers than the protein vaccine. Analysis of the ratio of IgG1 and IgG2a indicates that BALB/c mice immunized with recombinant protein vaccine with a Th-2 type immune response, as manifested by dominant IgG1 antibodies.

As mentioned above, the T cells could exist a much longer time than the antibodies in the recovered patients of SARS, indicating that cellular immunity responses should be studied carefully in designing SARS-CoV-2 vaccine. As the result shown (**Figure 4**), the mRNA vaccine performed better than the protein vaccine in inducing cellular immune response. Not only were more specific memory T cells observed in the mRNA vaccine group, but also the size of the spots was significantly larger than the protein vaccine and positive control groups as well, indicating that the mRNA vaccine strongly enhanced the ability of IFN-γ secretion in infection. These differences may be caused by the different antigen-presenting mechanisms of the two types of vaccine. For protein vaccines, the antigen proteins are enclosed into endocytic vesicles and presented on the cell surface by MHC-II (major histocompatibility complex class II) molecules to the CD4^+^ helper T cells (19). However, for the mRNA vaccines, after being transfected into antigen-presenting cells *via* endocytosis, the antigen proteins are translated and processed in cell cytosol, and in this way, not only is the MHC-II pathway activated after the secretion of antigen, but the MHC-I pathway is activated as well, leading to both CD4^+^ and CD8^+^ robust T cell responses (20).

In summary, this study compared the humoral and cellular responses of two major SARS-CoV-2 vaccine types, mRNA and recombinant vaccine. As both vaccines demonstrated highly immunogenic, still significant differences in response profiling exist. Specifically, mRNA vaccine can induce higher cellular immune responses than recombinant protein vaccine. To date, this is the first reported study to directly compare the immune responses of both immune arms between SARS-CoV-2 mRNA and recombinant protein vaccines. This work lays a strong foundation for optimizing better vaccines against SARS-CoV-2. To gain further understanding of their differences, viral challenging study in animal model is likely needed. Although safety concerns nowadays prevent us from pursuing such study using live viruses, we believe that challenging study can reveal more important details. In this regard, a clinical trial to compare them in human subjects may be even considered in the future.

## Materials and Methods

### mRNA Synthesis

The linearized DNA template, encoding codon-optimized RBD of SARS-CoV-2 B.1.617.2 variant and incorporating the 5’ and 3’ untranslated regions and a poly-A tail, was obtained from DNA plasmid A1009 (Tsingke) by using SapI endonuclease. The RBD-mRNA was produced by *in vitro* transcription using T7 RNA polymerase. The Gluc-encoding mRNA was constructed in the same way from DNA plasmid A1007.

### Preparation and Characterization of mRNA Lipid Nanoparticles

mRNA_LNPs were prepared in microfluidic mixing technology with the NanoAssemblr Benchtop (Precision NanoSystems Inc.). Specifically, DLin-MC3-DMA, 1,2-Dioleoyl-sn-glycero-3-PE (DOPE), cholesterol and DMG-PEG_2000_ were dissolved in ethanol in the molar ratio of 50:10:38.5:1.5, and mRNA was dissolved in physiological water. Both of them were injected into the mixer at a 1:3 volume and at a combined final flow rate of 10 mL/min (2.5 mL/min ethanol, 7.5 mL/min aqueous). Residual ethanol in the final mixture was then removed by dialysis. The preparation was performed in a sterile environment at room temperature. DLS and TEM were employed to confirm the formation of the mRNA_LNPs. All of the mRNA_LNPs were kept at 4°C for three weeks, and the size and PDI of them were measured by DLS.

### Expression of Recombinant RBD *In Vitro*

HEK 293T cells were seeded in 6-well cell culture plates (2 × 10^6^ cells/well) in KOP 293 medium. Six hours later, RBD_LNPs containing different amount of mRNA (0.3µg, 0.6 µg, and 1 µg) were added into cells. After being cultured at 37°C with 5% CO2 for 48 hours, cells were centrifuged at 1000 rpm for 10 minutes, and the supernatant were collected for further analysis.

### Animals

The animal studies were carried out at Beijing University of Chemical Technology, which were in strict accordance with the guidelines evaluated and approved by the ethics committee of University Animal Care and Use Committee and followed the international standards on animal welfare.

### Expression of Recombinant RBD *In Vivo*

Twelve mice were divided in groups of six randomly and administrated intramuscularly at 1 mg/kg RBD_LNPs or equivalent volume of PBS. The orbital blood was collected at different time point after administration (0 h, 6 h, 12 h, and 24 h). After being centrifuged at 2000 rpm for 15 minutes, the serum was collected and stored at -20°C for further analysis.

### Evaluation of RBD Expression *In Vivo* and *In Vitro*

A standard curve of different concentration of Delta variant RBD protein was established by ELISA. Briefly, a 96-well plate was coated with different concentration of RBD at 4°C overnight, and then the plate was washed three times with PBST and blocked with 2% Difco™ Skim Milk at 37°C for 1 h. After five washes with PBST, the plate was incubated with anti-RBD-hFc antibody at 37°C for 1 h. To develop the reaction, the plates were washed five times and incubated with horseradish peroxidase-conjugated antihuman IgG-Fc secondary antibody at 37°C for 1 h and washed five times. The reaction was visualized by TMB Single-Component Substrate solution and stopped with 2 N HCl. The absorbance at 450 nm (A450) was measured by ELISA plate reader. After constructing the standard curve of different concentration of RBD protein in GraphPad Prism 9, the supernatant or serum was analysis in the same ELISA protocol to quantify the expression of RBD *in vitro* and *in vivo*.

### Recombinant RBD Vaccine Candidate

Codon-optimized genes encoding residues 1-13, followed by 331-524, of SARS-CoV-2 spike protein were expressed in HEK 293 cell and purified from culture supernatant. Poly I:C-PLL was prepared in physiological water by adding the poly-L-lysine to poly I:C dropwise in a molar ratio of 0.5:1. The process was under magnetic stirring in a sterile environment. The lipid-based adjuvant was prepared with the NanoAssemblr Benchtop in a sterile environment at room temperature. In brief, the lipid components (DOTAP, DOPE, cholesterol and DMG-PEG were dissolved in ethanol in the molar ratio of 50:10:38.5:1.5) were dissolved in ethanol and the poly I:C-PLL was dissolved in physiological water, both of which were injected into the microfluidic mixer at a 1:3 volume and at a combined final flow rate of 10 mL/min. Residual ethanol in the final mixture was then removed by dialysis.

### Animal Vaccination and Sample Collection

Eighteen BALB/c mice were divided into three groups randomly (n=6). One group was vaccinated with 1µg/g mouse weight of recombinant RBD protein (in 100µl physiological water) in the presence of best adjuvant which we selected in our previous work; another group was vaccinated with 100µl RBD_LNPs which contain 5 µg modified RBD-encoding mRNA; the third group was administrated with 100µl PBS as the control. Mice were boosted with the same vaccine formulation or PBS after two weeks. Sera were collected at different time point as schedule (shown in **Figure 2A**) to assess SARS-CoV-2 RBD-specific antibody responses and *in vitro* neutralization assay. All groups of mice were sacrificed on day 35 after the first immunization, and splenocytes were collected to detect SARS-CoV-2 RBD-specific T-cell response.

### Evaluation of the Humoral Immune Response

ELISA was used to measure murine antibody responses induced by different vaccines. Briefly, ELISA plates were pre-coated with SARS-CoV-2 recombinant RBD protein overnight at 4°C, After three washes with PBST, serial dilution of mouse sera (from 1:1000 to 1:2048000) were added to plated and incubated at 37°C for 1 h. To develop the reaction, the plates were washed five times and incubated with horseradish peroxidase-conjugated secondary antibody (antimouse IgG, IgG1, or IgG2a) at 37°C for 1 h, and washed five times. The reaction was visualized by TMB Single-Component Substrate solution and stopped with 2 N HCl. The absorbance at 450 nm (A450) was measured by ELISA plate reader.

### *In Vitro* Neutralization Assay

Vero cells were plated in 96-well plates (2×10^5^ cells/well) and incubated overnight. Serial dilutions of serum were incubated with 650 TCID_50_ of the pseudovirus of SARS-CoV-2 B1.617.2 variant for 1 hour at room temperature before transfer to Vero cells. After 72 h of incubation, the supernatant was removed, and luciferase substrate was added. 2 minutes later, luciferase activity was measured and NT_50_ was defined as the serum dilution at which the relative light units (RLUs) were reduced by 50% compared with the virus control wells.

### Evaluation of Cytotoxic Immune Response

An ELISpot assay was used to evaluate cytotoxic immune response elicited by different vaccines. Briefly, on day 35 after the initial immunization, spleens from immunized mice were harvested and both grinded and filtered through 40 µm cell strainers. Splenocytes were collected and tested by IFN-γ ELISpot Kit. In brief, the plate was blocked using RPMI Medium 1640 (Solarbio, Beijing, China) containing 10% FBS and incubated for at least 30 minutes. Then, splenocytes collected from immunized mice were plated at 2.5×10^5^ cells/well, with overlapped peptide pool derived from SARS-CoV-2 RBD, RPMI Medium 1640 as negative control and Phytohaemagglutinin A (PHA) as positive control. After incubation at 37°C, 5% CO_2_ for 48 hours, the plate was washed with PBS for 4 times. Biotinylated anti-mouse IFN-γ antibody was added to each well and was incubated for 2 hours at room temperature. The reaction was visualized by AEC substrate solution, and the plate was read on CTL ELISPOT reader. The number and mean spot size of spot-forming cells (SFC) were recorded.

### Statistical Analysis

All statistical analysis were performed using GraphPad Prism 9.0 software (GraphPad Software). Statistical significance among different vaccination groups was analyzed by using two-way multiple ANOVA test, as specified in the figure legends. The values are presented as the means ± SEM unless otherwise noted.

## Data Availability Statement

The original contributions presented in the study are included in the article/**Supplementary Material**, further inquiries can be directed to the corresponding authors.

## Ethics Statement

The animal study was reviewed and approved by Beijing University of Chemical Technology.

## Author Contributions

YW conceived the ideas of research, prepared materials, analyzed the data, and wrote the manuscript. HZ and LM performed the animal surgery. FL and CY provided the lab resource and funding, supervised project, revised and edited the manuscript. All authors contributed to the article and approved the submitted version.

## Funding

This work was supported by the National Natural Science Foundation of China (Grant No. 82174531).

## Conflict of Interest

Author FL was employed by company Sun Yat-sen Biomedical Institute Limited. Author HZ and FL were employed by the company Sysvax Inc.

The remaining authors declare that the research was conducted in the absence of any commercial or financial relationships that could be construed as a potential conflict of interest.

## Publisher’s Note

All claims expressed in this article are solely those of the authors and do not necessarily represent those of their affiliated organizations, or those of the publisher, the editors and the reviewers. Any product that may be evaluated in this article, or claim that may be made by its manufacturer, is not guaranteed or endorsed by the publisher.

## References

[1] Hoffmann M Kleine-Weber H Schroeder S Kruger N Herrler T Erichsen S. SARS-CoV-2 Cell Entry Depends on ACE2 and TMPRSS2 and Is Blocked by a Clinically Proven Protease Inhibitor. Cell (2020) 181(2):271–80.e8. doi: 10.1016/j.cell.2020.02.052 32142651 PMC7102627

[2] Huang Y Yang C Xu XF Xu W Liu SW. Structural and Functional Properties of SARS-CoV-2 Spike Protein: Potential Antivirus Drug Development for COVID-19. Acta Pharmacol Sin (2020) 41(9):1141–9. doi: 10.1038/s41401-020-0485-4 PMC7396720 32747721

[3] Wang Y Wang L Cao H Liu C. SARS-CoV-2 S1 is Superior to the RBD as a COVID-19 Subunit Vaccine Antigen. J Med Virol (2021) 93(2):892–8. doi: 10.1002/jmv.26320 PMC7404424 32691875

[4] Verbeke R Lentacker I De Smedt SC Dewitte H. The Dawn of mRNA Vaccines: The COVID-19 Case. J Control Release (2021) 333:511–20. doi: 10.1016/j.jconrel.2021.03.043 PMC8008785 33798667

[5] Zhang NN Li XF Deng YQ Zhao H Huang YJ Yang G. A Thermostable mRNA Vaccine Against COVID-19. Cell (2020) 182(5):1271–83.e16. doi: 10.1016/j.cell.2020.07.024 32795413 PMC7377714

[6] Cao Y Gao GF. mRNA Vaccines: A Matter of Delivery. EClinicalMedicine (2021) 32:100746. doi: 10.1016/j.eclinm.2021.100746 33644722 PMC7889829

[7] Park KS Sun X Aikins ME Moon JJ. Non-Viral COVID-19 Vaccine Delivery Systems. Adv Drug Delivery Rev (2021) 169:137–51. doi: 10.1016/j.addr.2020.12.008 PMC7744276 33340620

[8] Elia U Rotem S Bar-Haim E Ramishetti S Naidu GS Gur D. Lipid Nanoparticle RBD-hFc mRNA Vaccine Protects Hace2 Transgenic Mice Against a Lethal SARS-CoV-2 Infection. Nano Lett (2021) 21(11):4774–9. doi: 10.1021/acs.nanolett.1c01284 34032435

[9] Teo SP. Review of COVID-19 mRNA Vaccines: BNT162b2 and mRNA-1273. J Pharm Pract (2021) 12:8971900211009650. doi: 10.1177/08971900211009650 33840294

[10] Bergmann CC Lane TE Stohlman SA. Coronavirus Infection of the Central Nervous System: Host-Virus Stand-Off. Nat Rev Microbiol (2006) 4(2):121–32. doi: 10.1038/nrmicro1343 PMC7096820 16415928

[11] Shin EC Sung PS Park SH. Immune Responses and Immunopathology in Acute and Chronic Viral Hepatitis. Nat Rev Immunol (2016) 16(8):509–23. doi: 10.1038/nri.2016.69 27374637

[12] Tang F Quan Y Xin ZT Wrammert J Ma MJ Lv H. Lack of Peripheral Memory B Cell Responses in Recovered Patients With Severe Acute Respiratory Syndrome: A Six-Year Follow-Up Study. J Immunol (2011) 186(12):7264–8. doi: 10.4049/jimmunol.0903490 21576510

[13] Ng OW Chia A Tan AT Jadi RS Leong HN Bertoletti A. Memory T Cell Responses Targeting the SARS Coronavirus Persist Up to 11 Years Post-Infection. Vaccine (2016) 34(17):2008–14. doi: 10.1016/j.vaccine.2016.02.063 PMC7115611 26954467

[14] Huber VC McKeon RM Brackin MN Miller LA Keating R Brown SA. Distinct Contributions of Vaccine-Induced Immunoglobulin G1 (IgG1) and IgG2a Antibodies to Protective Immunity Against Influenza. Clin Vaccine Immunol (2006) 13(9):981–90. doi: 10.1128/CVI.00156-06 PMC1563571 16960108

[15] Kipps TJ Parham P Punt J Herzenberg LA. Importance of Immunoglobulin Isotype in Human Antibody-Dependent, Cell-Mediated Cytotoxicity Directed by Murine Monoclonal Antibodies. J Exp Med (1985) 161(1):1–17. doi: 10.1084/jem.161.1.1 3918141 PMC2187540

[16] Takai T Li M Sylvestre D Clynes R Ravetch JV. FcR Gamma Chain Deletion Results in Pleiotrophic Effector Cell Defects. Cell (1994) 76(3):519–29. doi: 10.1016/0092-8674(94)90115-5 8313472

[17] Nimmerjahn F Ravetch JV. Divergent Immunoglobulin G Subclass Activity Through Selective Fc Receptor Binding. Science (2005) 310(5753):1510–2. doi: 10.1126/science.1118948 16322460

[18] Nimmerjahn F Bruhns P Horiuchi K Ravetch JV. FcgammaRIV: A Novel FcR With Distinct IgG Subclass Specificity. Immunity (2005) 23(1):41–51. doi: 10.1016/j.immuni.2005.05.010 16039578

[19] Stern LJ Santambrogio L. The Melting Pot of the MHC II Peptidome. Curr Opin Immunol (2016) 40:70–7. doi: 10.1016/j.coi.2016.03.004 PMC4884503 27018930

[20] Wadhwa A Aljabbari A Lokras A Foged C Thakur A. Opportunities and Challenges in the Delivery of mRNA-Based Vaccines. Pharmaceutics (2020) 12(2):102–29. doi: 10.3390/pharmaceutics12020102 PMC7076378 32013049

