# Supplementary material for: Comparison of Immune Responses Elicited by SARS-CoV-2 mRNA and Recombinant Protein Vaccine Candidates
Source: Front Immunol. 2022 May 19;13:906457. doi: 10.3389/fimmu.2022.906457 (PMC9161160; doi:10.3389/fimmu.2022.906457)
Supplement: Supplementary file 1 [file DataSheet_1.docx]

Supplementary Materials for

**Comparison of Immune Responses Elicited by SARS-CoV-2 mRNA and Recombinant Protein Vaccine Candidates**

Yixin. Wu,^1^ Huicong. Zhang,^2^ Liuxian. Meng,^1^ Fusheng. Li,^2,3^* Changyuan. Yu,^1^*

^1^ College of Life Science and Technology; Beijing University of Chemical Technology, Beijing 100029, China.

^2^ Research Department, Sysvax Inc, Beijing, 102600, China

^3^ Vaccine Division, Sun Yat-sen Biomedical Institute Limited, Hongkong, China.

*** Correspondence:**[fushengli@sysvax.com](mailto:fushengli@sysvax.com) (F. Li); [yucy@mail.buct.edu.cn](mailto:yucy@mail.buct.edu.cn) (Y.Yu)

**This PDF file includes:**

Fig. S1. **Characterization of Gluc-encoding mRNA LNPs**

Fig. S2. **The SARS-CoV-2-specific IgG antibody titers elicited by different dose mRNA vaccine**


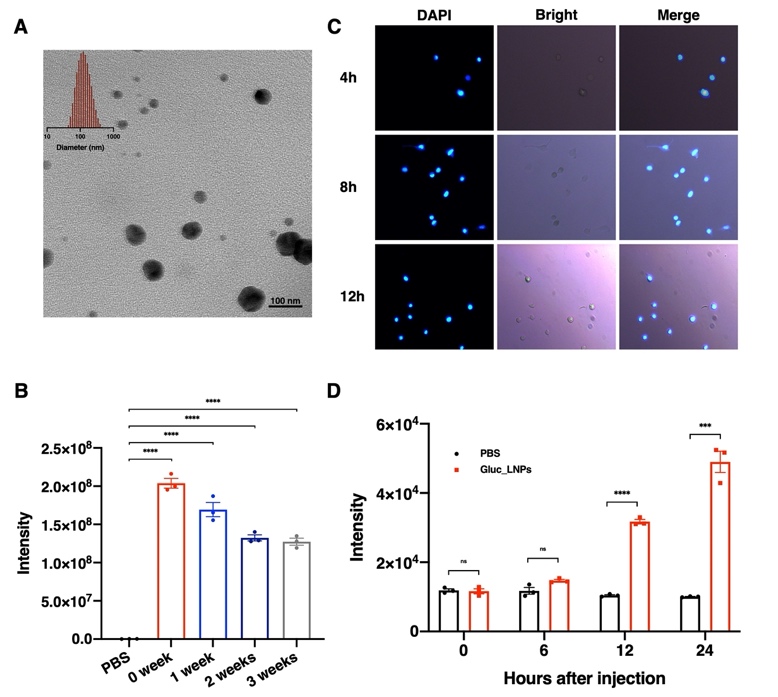


Fig. S1. Diameter and PDI each lipopolyplex. (A) TEM and DLS of Gluc-encoding mRNA LNPs. (B) The fluorescence intensity of cell supernatant after transfected with Gluc_LNPs which were stored at 4℃ for 0, 1, 2, and 3 weeks. (C) Gluc_LNPs which had been stored at 4℃ for two weeks were used to incubate DC 2.4 cells for 4 h, 8 h, and 12 h. The Gluc was examined by coelenterazine via the fluorescence microscope (D) Expression of Gluc in mice at different time point after the injection with Gluc_LNPs which had been stored at 4℃ for two weeks. The sera concentration of Gluc was examined by coelenterazine via fluorescence detection.

Fig. S2. The SARS-CoV-2-specific IgG antibody titers elicited by different dose mRNA vaccine.
